# The Effect of Mediterranean Diet on Thyroid Gland Activity

**DOI:** 10.3390/ijms25115874

**Published:** 2024-05-28

**Authors:** Iva Jureško, Nikolina Pleić, Ivana Gunjača, Vesela Torlak, Dubravka Brdar, Ante Punda, Ozren Polašek, Caroline Hayward, Tatijana Zemunik, Mirjana Babić Leko

**Affiliations:** 1 Department of Biology and Human Genetics, School of Medicine, University of Split, 21 000 Split, Croatia; 2 Department of Nuclear Medicine, University Hospital of Split, 21 000 Split, Croatia; 3 Department of Public Health, School of Medicine, University of Split, Šoltanska 2, 21 000 Split, Croatia; 4 Algebra University College, 10 000 Zagreb, Croatia; 5 MRC Human Genetics Unit, Institute of Genetics and Cancer, University of Edinburgh, Edinburgh EH4 2XU, UK; caroline.hayward@ed.ac.uk

**Keywords:** mediterranean diet, Mediterranean Diet Serving Score, thyroid, TSH, thyroid hormones, calcitonin

## Abstract

The main goal of this research was to determine whether there is a correlation between adherence to the Mediterranean diet (assessed by the Mediterranean Diet Serving Score (MDSS)) and parameters indicating thyroid gland activity, such as concentration of thyroid-stimulating hormone (TSH), thyroid hormones (free triiodothyronine (fT3), free thyroxine (fT4)), thyroglobulin (Tg), antibodies to thyroid proteins (thyroglobulin antibodies (TgAb) and thyroid peroxidase antibodies (TPOAb)), and calcitonin (CT) in plasma and serum samples. An additional objective was to investigate whether there are differences in the values of the MDSS among clinical groups (euthyroid individuals, euthyroid individuals with positive TgAb and/or TPOAb, and hypothyroid and hyperthyroid participants). This cross-sectional study included 4620 participants over 18 years of age from the islands of Korčula and Vis, and the mainland city of Split. The MDSS was assessed from a food frequency questionnaire (FFQ). MDSS values were significantly higher in females compared to males and showed a positive association with the age of the participants. There was no significant difference in the MDSS values among the examined clinical groups. In the group of subjects with euthyroidism, a significant positive association was found between fT3 and the MDSS, while in the group of subjects with subclinical hypothyroidism, a significant positive association was observed between the MDSS and both fT3 and fT4. CT levels were also positively associated with the MDSS. Considering the significant positive association of the MDSS and both fT3 and fT4 levels in patients with subclinical hypothyroidism, the results of this study could be used to create guidelines for selecting an appropriate, potentially protective diet for these patients.

## 1. Introduction

The Mediterranean diet is one of the healthiest dietary patterns today. It is based on the traditional diets of Mediterranean countries such as Croatia, Greece, Spain, and Italy [1]. It emphasizes the consumption of plant-based foods, including the daily intake of fruits, vegetables, whole grains, dairy products, and nuts. Olive oil, preferably extra virgin, is the primary source of fat in the Mediterranean diet. The consumption of animal-based foods is limited to several times a week, with a preference for fish, seafood, and white meat over red meat [2,3]. The intake of sweets is restricted, and moderate consumption of red wine is desirable [4]. Besides diet itself, an essential aspect of the Mediterranean diet is the lifestyle, which includes moderate physical activity, sufficient rest and sleep, cooking, seasonality, and the use of local and environmentally friendly food sources [2]. Studies have shown numerous benefits of the Mediterranean diet, including reduced body mass index (BMI), decreased prevalence of metabolic syndrome, type 2 diabetes, cardiovascular diseases, and certain types of cancer, as well as observed benefits for mental health [1,5].

To date, few studies have investigated the influence of the Mediterranean diet on thyroid gland activity [6,7]. This small endocrine gland located in the front of the neck just below the Adam’s apple is responsible for the production of thyroid hormones. The production of thyroid hormones is regulated by negative feedback at the level of the hypothalamus–pituitary–thyroid axis. Neurons in the paraventricular nuclei of the hypothalamus sense circulating levels of thyroid hormones (triiodothyronine (T3) and thyroxine (T4)) [8]. Thyroid hormones bind to thyroid hormone receptor isoform β2 (TRβ2) on these neurons, inducing the expression of the gene coding for thyrotropin-releasing hormone (TRH). When hormone levels are high, TRH gene expression is low, and vice versa. TRH released from the hypothalamus reaches the anterior pituitary via the hypothalamo-hypophyseal portal vessels. Binding to TRH receptors activates G-proteins within pituitary cells, leading to increased cyclic adenosine monophosphate (cAMP) levels and thyroid-stimulating hormone (TSH) release. TSH binds to receptors on follicular cells of the thyroid gland, activating intracellular cascades and increasing cAMP levels, leading to hormone secretion and glandular tissue growth [9]. The thyroid gland secretes two important hormones, T4 and T3, with T4 being the main product converted into the more active T3 in peripheral tissues. This complex feedback loop maintains a nearly constant hormone concentration in the bloodstream [10,11]. In the bloodstream, T3 and T4 are converted into their active forms, free T3 (fT3) and free T4 (fT4), which exhibit a higher affinity for binding to cellular receptors. The main site of hormone action is the cell nucleus, where their receptors are located either near DNA chains or bound to them. Thyroid hormone receptors act as transcription factors that activate numerous genes. Thyroid hormones are essential for proper growth, development, metabolic processes, energy expenditure, fat and carbohydrate metabolism, and reproductive function [12,13]. In addition to thyroid hormones, through parafollicular or C cells, the thyroid gland also produces the hormone calcitonin (CT), whose main function is to regulate calcium levels in the body, primarily by reducing the amount of calcium released from bones into the bloodstream [14].

The main objective of this study was to determine whether there is a correlation between the Mediterranean Diet Serving Score (MDSS), which is used for the assessment of adherence to the Mediterranean diet and parameters indicating thyroid gland activity, such as plasma levels of TSH, thyroid hormones (fT3, fT4), thyroglobulin (Tg), antibodies to thyroid proteins (thyroglobulin antibodies (TgAb) and thyroid peroxidase antibodies (TPOAb)), and serum levels of CT. An additional aim was to ascertain whether there are differences in MDSS values between euthyroid, euthyroid with positive TgAb and/or TPOAb values, hypothyroid, and hyperthyroid individuals.

## 2. Results

This study included 4620 participants. Out of the total of 4620 participants, the MDSS was determined for 4495 participants, with 889 of them (19.8%) shown to adhere to the Mediterranean diet (MDSS ≥ 13.5). The values of the MDSS, biochemical parameters, and demographic data are presented in Table 1. The values of the MDSS were significantly higher in the group of female participants compared to male participants (t = 9.779, df = 4493, *p <* 0.001). The values of the MDSS were positively associated with the age of the participants (β = 0.220, SE = 0.062, *p <* 0.001). Therefore, all analyses were adjusted for the influence of sex and age.

### 2.1. Comparison of Mediterranean Diet Serving Score Values between Clinical Groups

**Figure 1:**
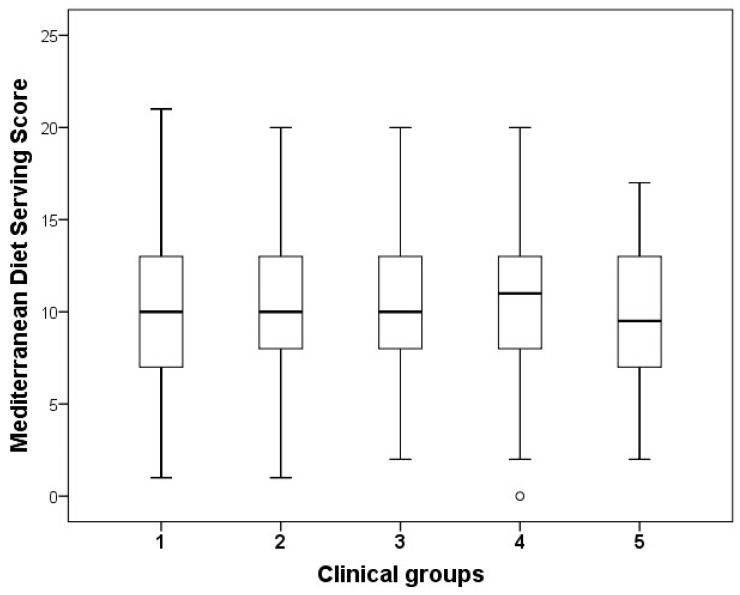
Comparison of Mediterranean Diet Serving Score values between clinical groups: euthyroidism (1); euthyroidism with positive antibodies (2); subclinical hypothyroidism (3); clinical hypothyroidism; and (4) subclinical and clinical hyperthyroidism (5).

There was no statistically significant difference in the values of the MDSS between the five investigated groups (participants with euthyroidism, participants with euthyroidism and positive antibodies, participants with subclinical hypothyroidism, participants with clinical hypothyroidism, and participants with subclinical and clinical hyperthyroidism) (F = 1.081, *p =* 0.364, Figure 1).

### 2.2. Correlations of TSH, Thyroid Hormones, Antibodies to Thyroid Proteins, and Calcitonin Values with the Mediterranean Diet Serving Score

**Figure 2:**
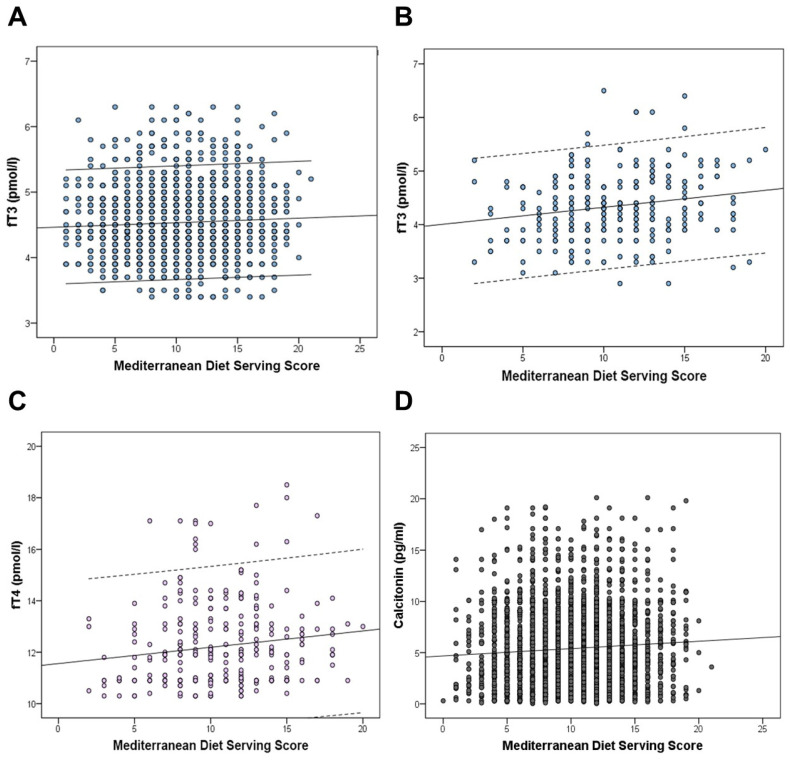
Positive correlation between the Mediterranean Diet Serving Score and (**A**) fT3 values in the group of subjects with euthyroidism, (**B**) fT3 values in the group of subjects with subclinical hypothyroidism, (**C**) fT4 values in the group of subjects with subclinical hypothyroidism, and (**D**) CT values across all subjects.

Values of TSH, thyroid hormones, Tg, and antibodies to thyroid proteins were correlated with the MDSS values in each clinical group. In the group of subjects with euthyroidism, a significant positive correlation was observed between fT3 and the MDSS (r = 0.058, *p =* 0.008; Figure 2). This was also confirmed after correction for the influence of sex and age, i.e., linear regression showed a significant positive association between the MDSS and fT3 values (β = 0.065, SE = 0.002, *p =* 0.001, MDSS = independent variable, fT3 = dependent variable). In the group of subjects with euthyroidism, no significant correlation was found between the MDSS and TSH (r = −0.033, *p =* 0.077), fT4 (r = 0.027, *p =* 0.145), or Tg levels (r = 0.028, *p =* 0.180).

In the group of subjects with subclinical hypothyroidism, a significant positive correlation was found between the MDSS and fT3 (r = 0.189, *p =* 0.006; Figure 2) and fT4 (r = 0.140, *p =* 0.014; Figure 2). After correction for the influence of sex and age, a positive association was also confirmed between the MDSS and fT3 (β = 0.228, SE = 0.010, *p <* 0.001) and fT4 (β = 0.171, SE = 0.027, *p =* 0.005) (MDSS = independent variable, thyroid hormones = dependent variables). In the group of subjects with subclinical hypothyroidism, no significant correlation was found between the MDSS and TSH (r = 0.052, *p =* 0.364), Tg (r = 0.013, *p =* 0.827), TgAb (r_S_ = 0.081, *p =* 0.155), or TPOAb values (r_S_ = 0.116, *p =* 0.252).

In the group of subjects with euthyroidism with positive antibodies, no significant correlation was found between MDSS and TSH (r = −0.012, *p =* 0.748), fT3 (r = −0.020, *p =* 0.589), fT4 (r = −0.046, *p =* 0.209), Tg (r = 0.046, *p =* 0.268), TgAb (r_S_ = 0.081, *p =* 0.168), or TPOAb values (r_S_ = 0.064, *p =* 0.083).

In the group of subjects with hyperthyroidism (subclinical + clinical hyperthyroidism), no significant correlation was found between the MDSS and TSH (r = 0.009, *p =* 0.955), fT3 (r = 0.170, *p =* 0.275), fT4 (r = 0.043, *p =* 0.779), Tg (r = −0.296, *p =* 0.095), TgAb (r_S_ = 0.146, *p =* 0.344), or TPOAb values (r_S_ = −0.046, *p =* 0.766).

CT levels were correlated with the MDSS values in all subjects with determined CT levels, regardless of the presence of autoimmune thyroid disease (overall 3514 subjects). There was a positive correlation between CT levels and MDSS values (r = 0.070, *p <* 0.001). This was also confirmed after correction for the influence of sex and age (β = 0.091, SE = 0.019, *p <* 0.001, Figure 2) (MDSS = independent variable, CT = dependent variable). Since previous studies indicated that smoking status [15] and renal dysfunction [16] can influence CT levels, we additionally analyzed CT levels in relation to these factors. Our analysis revealed that CT levels did not differ significantly between participants with and without renal disease (*p =* 0.417). Additionally, smoking status and serum uric acid levels, a factor associated with renal function, were not significant predictors of CT levels (*p =* 0.331 and *p =* 0.687, respectively). However, serum creatinine levels, another factor associated with renal function, were a significant negative predictor of CT levels (*p =* 0.037). The results of the association between CT levels and MDSS values remained significant after the analyses were controlled individually for the influence of smoking (β = 0.092, SE = 0.019, *p <* 0.001), and factors associated with renal function (the presence of renal disease (β = 0.092, SE = 0.019, *p <* 0.001), serum creatinine levels (β = 0.089, SE = 0.019, *p <* 0.001), and serum uric acid levels (β = 0.091, SE = 0.019, *p <* 0.001)). Importantly, when controlling the analysis for all aforementioned variables, the association between CT and MDSS still remained significant (β = 0.093, SE = 0.019, *p <* 0.001).

## 3. Discussion

The aim of this cross-sectional study, which included 4620 participants (MDSS determined for 4495 participants), was to determine whether there was an association between the MDSS and parameters reflecting thyroid gland activity (TSH, fT4, fT3, Tg, TgAb, TPOAb, and CT levels measured in participants’ plasma and serum samples). It was found that an unexpectedly small number of individuals (19.8%) in Dalmatia adhere to the Mediterranean diet (MDSS ≥ 13.5), considering the geographical location and traditional diet. The average MDSS value was only 10 out of a possible 24 points. It was shown that women adhere more to the Mediterranean diet than men and that the MDSS increases with the age of the participants. Participants were divided into five clinical groups: participants with euthyroidism, participants with euthyroidism with positive antibodies, participants with subclinical hypothyroidism, participants with clinical hypothyroidism, and participants with subclinical and clinical hyperthyroidism. The MDSS did not significantly differ between the five clinical groups, but the results of this study showed a significant positive correlation between MDSS and fT3 levels in the group of participants with euthyroidism and a significant positive correlation between MDSS and both fT3 and fT4 levels in patients with subclinical hypothyroidism, while no statistically significant correlations were observed in other groups. No associations were observed between MDSS and TSH, Tg, TgAb, and TPOAb levels in any of the groups. CT levels were positively associated with MDSS.

To date, only a small number of studies have investigated the association between the Mediterranean diet and thyroid gland activity. In a study involving 324 healthy obese or overweight participants, Zupo et al. observed significantly lower levels of fT3 and fT4 in participants adhering to the Mediterranean diet compared to those who did not. TSH values were unchanged between these two groups of participants [6]. In their study, Zupo et al. used the PREDIMED (PREvención con DIeta MEDiterránea) questionnaire to assess adherence to the Mediterranean diet, whereas our study used the MDSS. Additionally, Zupo et al. included a significantly smaller number of participants in their study (324 compared to 4495 in our study); their research was conducted in obese participants, whereas our study was not limited to this group. It is well known that BMI affects TSH and thyroid hormone levels [17]. Therefore, all of this could explain the contradictory results observed in our study and the study of Zupo et al. [6]. In a study from 2022, Barrea et al. assessed adherence to the Mediterranean diet using the PREDIMED questionnaire and found that poorer adherence was associated with a higher prevalence of thyroid nodular disease, especially nodules at high risk for malignant disease [7]. This study included 794 participants, with confirmed thyroid nodular disease in 391 participants. After reviewing the available literature, Barrea et al. confirmed that the Mediterranean diet is associated with a reduced risk of thyroid nodular disease and thyroid carcinoma and has numerous benefits for reproductive health and the treatment of neuroendocrine tumors due to its anti-inflammatory and antioxidant effects [18]. In a longitudinal study from 2022, Llaha et al. followed 450,064 men and women from nine European countries for 14 years, of whom 712 developed differentiated thyroid carcinoma. Using the relative Mediterranean diet score (rMED score) and adjusted relative Mediterranean diet score (arMED score), they assessed how well participants adhered to the Mediterranean diet. The results showed that high adherence to the Mediterranean diet was not associated with the development of differentiated thyroid carcinoma [19].

After reviewing the available literature, Ruggeri et al. concluded that current evidence suggests that the Mediterranean diet, with an emphasis on plant-based foods, may have a potentially protective effect against the development of autoimmune thyroid diseases, while the Western high-calorie diet rich in animal fats, salt, and refined foods promotes autoimmunity by affecting the immune system, gut microbiota, increasing oxidative stress, and promoting inflammation [20]. Numerous studies have shown that individual components of the Mediterranean diet have a beneficial effect on the thyroid gland. Ruggeri et al. confirmed, through a review of the available literature, that blue fish rich in omega-3 fatty acids have a protective effect against the development of autoimmune thyroiditis [20]. Brdar et al. demonstrated that the consumption of blue and white fish and seafood significantly increases fT3 and fT4 levels [21]. Extra virgin olive oil acts protectively on thyroid tissue and stimulates thyroid function in euthyroid and hypothyroid animals [22]. Moderate alcohol consumption, one of the main principles of the Mediterranean diet, reduces the risk of developing Hashimoto’s thyroiditis and thyroid cancer [23]. Seafood and fish are rich in selenium, iodine, zinc, and iron and have a protective effect on the development of autoimmune thyroid diseases [24]. The Mediterranean diet seems to have numerous positive effects on the prevention of thyroid diseases, with a focus on autoimmune diseases and carcinomas.

The influence of the Mediterranean diet on CT levels was not previously analyzed. The positive association of CT levels with MDSS values observed in this study could be explained by the fact that subjects with higher adherence to the Mediterranean diet have a higher dietary calcium intake, mostly consumed through the intake of dairy products [25]. This can lead, in turn, to the increase in CT levels in the blood since CT is responsible for lowering calcium blood levels [26].

The limitation of this study is that it is a cross-sectional study, so we cannot directly infer a cause-and-effect relationship between adherence to the Mediterranean diet and parameters of thyroid activity and markers of autoimmune thyroid diseases. Additionally, collecting dietary data required participants to recall when filling out the questionnaire; thus, there is a possibility of recall bias. Finally, in the questionnaire on dietary habits, participants themselves estimate how frequently they consume certain foods, and their intake of foods is not directly quantified.

The greatest strength of this research is the large number of participants from the Dalmatia region, which, as part of the Mediterranean Basin, is a traditional place of the Mediterranean diet and lifestyle, making it an ideal area for studying the frequency of adherence to the Mediterranean diet and its effect on thyroid function.

In conclusion, our study showed a positive association between the MDSS and fT3 levels in the group of participants with euthyroidism, and a positive association between MDSS and both fT3 and fT4 levels in patients with subclinical hypothyroidism. Additionally, CT levels were positively associated with MDSS. There was no significant difference in the values of the MDSS between euthyroid participants and participants with hypothyroidism or hyperthyroidism. Additionally, the values of the MDSS did not show a significant association with TSH values and antibodies to thyroid proteins. Future studies should conduct randomized controlled clinical trials to better elucidate the potential protective role of the Mediterranean diet in the development of autoimmune thyroid diseases and thyroid cancer. Furthermore, considering that participants with higher values of the MDSS had higher fT3 and fT4 values, the results of this study could be used to create guidelines for selecting an appropriate, potentially protective diet for patients with subclinical hypothyroidism.

## 4. Materials and Methods

### 4.1. Research Organization and Participants

This research was designed as a cross-sectional study. Participants were recruited through the “10,001 Dalmatians” project [27]. Initially, 4848 participants over the age of 18 were included, but those taking thyroid medication or those who had undergone thyroid surgery were excluded, resulting in a final number of 4620 participants. The participants were from the Dalmatia region (2811 from the island of Korčula, 1025 from the island of Vis, and 1012 from the mainland city of Split). In the comparison of CT levels with MDSS, those participants who had a history of malignant tumors, underwent thyroid surgery, or had increased parathyroid hormone levels (overall 309 patients) were excluded from the study (resulting in a total of 3514 participants with determined CT levels). Each participant provided written informed consent, and the research protocol was approved by the Ethics Committee of the School of Medicine, University of Split (No: 2181-198-03-04-14-0031 and 2181-198-03-04-19-0022).

### 4.2. Biochemical Measurements

Blood samples were collected in the morning hours (from 7 to 9 a.m.) after overnight fasting. The concentrations of TSH, fT3, fT4, Tg, TgAb, and TPOAb in plasma were determined by immunoassay using the fully automated “Liaison Biomedica Chemiluminescence Analyzer” device (DiaSorin, Saluggia, Italy), while serum CT levels were determined by the radio-immunoassay method using the “Scintillation counter liquid samples” (Capintec, Florham Park, NJ, USA) at the Radiobiochemical Laboratory of the Clinical Department of Nuclear Medicine, Clinical Hospital Center in Split. The reference ranges for the analyzed parameters were as follows: TSH, 0.3–3.6 mIU/L; fT3, 3.39–6.47 pmol/L; fT4, 10.29–21.88 pmol/L; Tg, 0.2–50 ng/mL; TgAb, 5–100 IU/mL; TPOAb, 1–16 IU/mL; and CT, 1.2–10.9 pg/mL.

### 4.3. Clinical Groups of Participants

The participants were divided into five clinical groups: individuals with euthyroidism, individuals with euthyroidism with positive antibodies, individuals with subclinical hypothyroidism, individuals with clinical hypothyroidism, and individuals with subclinical and clinical hyperthyroidism. Due to the small number of participants, individuals with subclinical hyperthyroidism and clinical hyperthyroidism were grouped together for statistical analysis. Euthyroidism is defined as a condition in which the levels of TSH, fT3, and fT4 hormones are within the normal range, with no presence of positive antibodies. Participants who had normal values for all three hormones but had positive antibodies (TPOAb and/or TgAb) are classified into the euthyroidism group with positive antibodies. Subclinical hypothyroidism is defined as elevated TSH levels (>3.6 mIU/L) with normal fT3 and fT4 values, while clinical hypothyroidism is defined as elevated TSH (>3.6 mIU/L), low fT3 (<6.47 pmol/L), and low fT4 (<10.29 pmol/L). Subclinical hyperthyroidism is defined as low TSH levels (<0.3 mIU/L) with normal fT3 and fT4 values, while clinical hyperthyroidism is defined as low TSH (<0.3 mIU/L), high fT3 (>3.39 pmol/L), and high fT4 (>21.88 pmol/L). The cutoff values for TSH used in these definitions are those specified in the test kit instructions.

### 4.4. Assessment of Mediterranean Diet Adherence

We used a food frequency questionnaire (FFQ) to assess nutrient consumption. The questionnaire consisted of 56 questions on the consumption of various foods and beverages. Each question had 6 possible answers on the frequency of food consumption (1 = every day, 2 = 2 or 3 times a week, 3 = once a week, 4 = once a month, 5 = rarely, or 6 = never). The exceptions were 4 questions on fat consumption with three possible answers (1 = always, 2 = sometimes, and 3 = never).

We used the MDSS to assess adherence to the Mediterranean diet. According to the MDSS, we created 14 food categories which included vegetables, fruit, olive oil, cereals, dairy products, nuts, potatoes, eggs, fish, legumes, white meat, red meat, fermented beverages, and sweets [3]. Kolčić et al. used the same FFQ to assess adherence to the Mediterranean diet [1]. Thus, we were guided by their approach, with some modifications, in accordance with Marendić et al. [1,3]. Participants who consumed 1–2 servings of cereals, 1–2 servings of fruits, ≥2 servings of vegetables, and 1 serving of olive oil daily gained 3 points (for each of the listed categories). Participants who consumed 2 servings of dairy products and 1–2 servings of nuts daily gained 2 points (for each of the listed categories). Additionally, those who consumed ≥2 servings of legumes, ≥2 servings of fish and shellfish, 2 servings of white meat, 2 servings of red meat, 2–4 servings of eggs, ≤2 servings of sweets, and ≤3 servings of potatoes per week gained 1 point (for each of listed categories). Additionally, those who drank 1–2 glasses of wine per week gained 1 point [3]. According to Kolčić et al., we gave 0 points to those participants who exceeded the aforementioned guidelines for potatoes, meat, sweets, eggs, and wine [1]. Additionally, participants who did not reach the aforementioned guidelines in other categories received 0 points [1].

The FFQ included five questions on the consumption of vegetables (tomatoes, leafy, cruciferous, canned or pickled, and root vegetables). Participants who consumed at least 2 types of vegetables or 1 type of vegetables daily (plus the consumption of 2 other types of vegetables 2–3 times per week) gained 3 points. The FFQ included four questions on the consumption of cereals (wholegrain bread, white bread, muesli, pasta, or rice); those who consumed at least 1 type of cereal daily gained 3 points. The FFQ included two questions on fruits (fresh and dried); those who consumed at least 1 type of fruit daily gained 3 points. The FFQ included five questions on the consumption of dairy products (milk, yogurt, hard cheese, cottage cheese, and sour cream), and those who consumed at least 2 types of dairy products or 1 type of dairy product daily (plus the consumption of two other types of dairy products 2–3 times per week) gained 2 points. FFQ included four questions on the consumption of fish (white, blue, seafood, squid, and octopus), and those who consumed at least 1 type of fish 2–3 times per week gained 1 point. Additionally, the FFQ included two questions on the consumption of white meat (chicken and turkey), four questions on the consumption of red meat (pork, veal, beef, and lamb), six questions on the consumption of sweets (cakes, chocolate, cookies, bonbons, refreshing non-alcoholic drinks, and cedevita), and three questions on the consumption of wine (white wine, red wine, and bevanda).

### 4.5. Statistical Analysis

The values of the MDSS were compared between euthyroid individuals, euthyroid individuals with positive antibodies, individuals with subclinical and clinical hypothyroidism, and individuals with subclinical and clinical hyperthyroidism using one-way analysis of variance (ANOVA). Differences in the values of the MDSS between the two groups were analyzed using the post hoc Scheffé test. The association of the MDSS with the values of TSH, thyroid hormones (fT3 and fT4), Tg, antibodies to thyroid proteins (TgAb and TPOAb), and CT was analyzed using correlation (Pearson and Spearman correlation coefficients). Additionally, these analyses were adjusted for the influence of sex and age using linear regression (parameters indicating thyroid gland activity (TSH, fT4, fT3, Tg, TgAb, TPOAb plasma levels, and CT serum levels) = dependent variables, MDSS = independent variable). Before applying linear regression, regression assumptions (normality of residuals, linearity of data, and homoscedasticity) were tested using diagnostic plots. All variables were normally distributed, except for TSH, TgAb, and TPOAb, which exhibited right-skewed distribution, following an approximately log-normal distribution. Thus, before analysis, TSH, TgAb, and TPOAb values were logarithmically transformed.

Statistical analysis was performed using R software (R Foundation for Statistical Computing, Vienna, Austria), with a significance level of α = 0.05.

## Figures and Tables

**Table 1:** Clinical characteristics of the participants.

Variable	Values *
Sex	
Female	2792 (60.4%)
Male	1828 (39.6%)
Age	55 (42–66)
Mediterranean Diet Serving Score (MDSS)	10 (8–13)
TSH (mIU/L)	1.6 (1.1–2.5)
fT4 (pmol/L)	12.9 (11.9–14.1)
fT3 (pmol/L)	4.4 (4.2–4.8)
Tg (ng/mL)	10.1 (5.5–16.8)
TgAb (IU/mL)	8.5 (5–19.1)
TPOAb (IU/mL)	4.4 (1.7–11.5)
CT (pg/mL)	4.7 (2.4–7.9)
Clinical groups	
Euthyroid participants	2939 (70.5%)
Euthyroid participants with positive antibodies	747 (17.9%)
Subclinical hypothyroidism	313 (7.5%)
Clinical hypothyroidism	127 (3.1%)
Subclinical and clinical hyperthyroidism	45 (1%)

* Values of the investigated variables are presented as the median (25th–75th percentile) or absolute frequency (relative frequency). CT, calcitonin; fT3, free triiodothyronine; fT4, free thyroxine; Tg, thyroglobulin; TgAb, thyroglobulin antibodies; TPOAb, thyroid peroxidase antibodies; TSH, thyroid-stimulating hormone.

## Data Availability

The data presented in this study are available on request from the corresponding author.

## Abbreviations

BMI, body mass index; cAMP, cyclic adenosine monophosphate; CT, calcitonin; FFQ, food frequency questionnaire; fT3, free triiodothyronine; fT4, free thyroxine; MDSS, Mediterranean Diet Serving Score; T3, triiodothyronine; T4, thyroxine; Tg, thyroglobulin; TgAb, thyroglobulin antibodies; TPOAb, thyroid peroxidase antibodies, TRH, thyrotropin-releasing hormone; TRβ2, thyroid hormone receptor isoform β2; TSH, thyroid-stimulating hormone.
